# Fluorescent Cascade and Direct Assays for Characterization of RAF Signaling Pathway Inhibitors

**DOI:** 10.2174/1875397300801010043

**Published:** 2008-02-25

**Authors:** Kevin R Kupcho, Rica Bruinsma, Tina M Hallis, David A Lasky, Richard L Somberg, Tammy Turek-Etienne, Kurt W Vogel, Kristin G Huwiler

**Affiliations:** Invitrogen Corporation, 501 Charmany Drive, Madison, WI 53719, USA

**Keywords:** Cascade assay, Direct Assay, C-RAF, B-RAF, B-RAF V599E, MEK1, ERK2, FRET, TR-FRET.

## Abstract

RAF kinases are part of a conserved signaling pathway that impacts cell growth, differentiation, and survival, and RAF pathway dysregulation is an attractive target for therapeutic intervention. We describe two homogeneous fluorescent formats that distinguish RAF pathway inhibitors from direct RAF kinase inhibitors, using B-RAF, B-RAF V599E, and C-RAF. A Förster-resonance energy transfer (FRET) based method was used to develop RAF and MEK cascade assays as well as a direct ERK kinase assay. This method uses a peptide substrate, that is terminally labeled with a FRET-pair of fluorophores, and that is more sensitive to proteolysis relative to the phosphorylated peptide. A second time-resolved FRET-based assay using fluorescently labeled MEK substrate was used to detect direct inhibitors of RAF kinase activity. The cascade assays detect compounds that interact with activated and unactivated kinases within the recapitulated RAF pathway, and the direct assays isolate the point of action for an inhibitor.

## INTRODUCTION

Mitogen-activated protein kinase (MAPK) signaling pathways consist of highly conserved Ser/Thr kinases that form a critical link between extracellular stimuli and intracellular response. MAPK signaling pathways mediate numerous cellular activities, including proliferation, movement, differentiation, and apoptosis. There are several unique MAPK signaling pathways, but each propagates its signal *via *the sequential phosphorylation of a three kinase core: the most upstream kinase (MAPK kinase kinase, MAP3K) activates the middle kinase (MAPK kinase, MAP2K) *via *phosphorylation, which in turn phosphorylates/activates the terminal kinase (MAPK). The activated MAPK can then phosphorylate its target substrates, which include transcription factors [1].

One of the most highly studied MAPK pathways is the extracellular signal-regulated kinase (ERK1/2) pathway, which transmits its signals through a series of activation steps involving receptor tyrosine kinases (RTKs), Ras, RAF (MAP3K), MEK (MAP2K), and ERK (MAPK). The hyper-activation of this pathway is associated with ~30% of human cancers [2-4]. Numerous factors can contribute to ERK pathway activation, including the over-expression of RTKs and mutations within the RTK or Ras families [5-7]. Recently, mutations in B-RAF have been identified in human cancers that can contribute to ERK pathway activation, and one specific mutation (V599E), was found to have the highest incidence overall and accounts for ~90% of B-RAF mutations found in melanoma and thyroid cancer [8]. (Note: ****In this paper, we will use the original numbering for B-RAF; however, the V599E mutation is actually V600E and we refer the reader to the Note within [8] for a detailed description).

Use of B-RAF V599E kinase for both *in vitro* and **in vivo** experimentation has revealed it to be an oncogene that causes a ~500-fold increase in kinase activity **in vitro**, proliferation of cells expressing the mutant, and **in vivo** transformation of these cells within nude mice [8-12]. Reduction of mutant B-RAF levels in melanoma cells *via *RNA interference decreased ERK activity and cell proliferation [13]. Comparison of B-RAF V599E and wild-type (WT) kinase domain structures suggests that the inactive WT conformation is stabilized through interactions between the glycine-rich loop and the activation segment. V599 is found within this segment, and mutation to glutamate disrupts this interaction and allows the mutant to assume an active conformation [9].

RAF kinases play a role in tumorigenesis, and are associated with tumor metastasis, radiation and chemo-resistance, and angiogenesis [14]. Tumor-induced angiogenesis is the process by which tumors recruit the development of new blood vessels from the existing endothelial vasculature. These new blood vessels are critical if a tumor is to grow more than a few millimeters in diameter [15, 16]. A key step in the angiogenic process initiated by tumors is the secretion of growth and angiogenic factors, such as vascular endothelial growth factor (VEGF), basic fibroblast growth factor (bFGF), and platelet derived growth factor beta (PDGF-β ). These factors signal through RTKs on endothelial cells and ultimately through the RAF pathway. Sorafenib (Bayer, BAY-43-9006) is a compound that is approved for the treatment of advanced renal cell carcinoma, and is the first multikinase inhibitor that targets B-RAF, B-RAF V599E, and C-RAF. Other kinases that Sorafenib inhibits play a role in mediating angiogenic responses (including VEGF receptor -2 & -3, PDGF-beta receptor) and tumor growth (including FLT3 and c-Kit) [14, 17]. Two other compounds that inhibit the RAF pathway *via *an allosteric inhibition of MEK are PD-0325901 (Pfizer) and ARRY-142886 (AZD6244, Array and AstraZeneca) [14, 18, 19]. Allosteric kinase inhibitors are typically referred to as either type II or type III, where the compound’s binding site on the kinase partially overlaps with the ATP binding site for type II inhibitors, or is independent of the ATP binding site for type III inhibitors. Both PD-0325901 and ARRY-142886 inhibit MEK1 using an ATP non-competitive (type III) mode of interaction and have progressed into clinical trials [14, 19]. Thus, the identification of new compounds that inhibit the RAF signaling pathway may hold clinical benefit.

To address the need for simple, non-radiometric assays capable of identifying and characterizing inhibitors of the RAF signaling pathway, we have developed a series of fluorescent assays suitable for high-throughput screening that use either Förster resonance energy transfer (FRET) or time-resolved (TR) FRET. We will highlight the development of direct and cascade assays for the RAF-MEK-ERK pathway using C-RAF, B-RAF, and B-RAF V599E. Using reference compounds, we demonstrate how these assays can be used to biochemically interrogate the RAF pathway and identify ATP competitive and non-competitive inhibitors. In addition, we show how the FRET assays can be applied for profiling using greater than 230 kinases.

## MATERIALS AND METHODS

### Reagents and General Data Analysis

The FRET peptides were synthesized as a set containing a non-phosphorylated peptide substrate and phophorylated control peptide. The FRET peptides used for the RAF and MEK cascade assays as well as the ERK direct assay were part of the Z’-LYTE™ kinase assay kit- Ser/Thr 3 (invitrogen); this FRET peptide is derived from the ERK consensus phosphorylation site of PX(S/T)P found within myelin basic protein; other FRET peptides used were also from invitrogen. All active and unactivated kinases used in this study were from Invitrogen. The following kinases were all full-length: B-RAF, unactive MEK1, active MEK1, unactive-fluorescein-labeled MEK1, active ERK2, and unactive ERK2. B-RAF V599E and C-RAF consisted of the catalytic domains only, amino acids 416-766 for B-RAF V599E and 306-648 for C-RAF. C-RAF also contained two point mutations that rendered it constitutively active (Y340D and Y341D). Staurosporine was purchased from Sigma while GW5074, ZM336372, U0126, and PD98059 were from BioMol. Unless otherwise noted, all data were plotted and analyzed using GraphPad Prism software (GraphPad Software, Inc., San Diego, CA). Z’ values were calculated according to the following equation [20].

$$Z^{'}=1-\frac{3\left(\sigma_{c^{+}}+\sigma_{c^{-}}\right)}{\mu_{c^{+}}+\mu_{c^{-}}}$$

where σ_c+_ and σ_c-_ are the standard deviations of positive and negative control wells, respectively, while µ_c+_ and µ_c-_ are the mean values for the positive and negative control wells, respectively. 

### FRET-Based Kinase Assays 

Direct FRET-based kinase assays were performed in 384-well low-volume plates (Corning #3676) using 2 µM substrate and 100 µM ATP in a 10 µL volume. The buffer consisted of 50 mM HEPES pH 7.5, 10 mM MgCl_2_, 1 mM EGTA, 0.01% Brij-35. Kinase reactions were typically initiated by the addition of either ATP or the active kinase and allowed to proceed for 1 hour at room temperature (RT). Following the 1 hour kinase assay, 5 µL of development reagent was added and the development reaction allowed to progress for 1 hour at RT, essentially as described previously [21]. The reactions were then read on a Tecan Safire™ fluorescent plate reader (Tecan, Durham, NC) using an excitation wavelength of 400 nm (12 nm bandpass) and emission wavelengths of 445 nm (12 nm bandpass) and 520 nm (12 nm bandpass). Inhibitor titrations were performed in quadruplicate as follows: 2.5 µL of the inhibitor stock was added to a 5 µL solution of active kinase and FRET substrate, followed by 2.5 µL of ATP to start the assay. For inhibitor profiling studies using 1 µM or 10 µM BAY-43-9006, FRET-based direct kinase assays were conducted as described above with the following exceptions: (1) the final ATP concentration was equal to the ATP K_m_ apparent (ATP K_m _^app^) determined for each kinase in the direct FRET-based assay; and (2) kinase buffer composition and pH were optimized for each kinase.

FRET-based cascade assays utilizing multiple kinases in the pathway were conducted in a manner similar to that described above for the direct kinase assays, with the following exceptions. For double (MEK-ERK) and triple (RAF-MEK-ERK) cascade assays, it was necessary to first optimize the concentration of the unactivated kinase(s) prior to optimizing the concentration of the active kinase; this procedure is explained in the Results section. All cascade kinase reactions were conducted at a single ATP concentration of 100 µM. For the double and triple cascade assays, the amount of unactivated ERK2 in the 10 µL kinase reaction was ~140 nM (or 10 µg/ml). For the three triple cascade assays, the amount of unactivated MEK1 in the 10 µL kinase reaction was ~ 20 nM (or 1 µg/ml). The concentrations of active kinases in the double and triple cascade assays were selected to yield ~10 - 50% phosphorylation in the absence of inhibitor, as described in the Results (MEK1, ~4.2 nM; B-RAF, ~57 pM; B-RAF V599E, ~10.4 pM; C-RAF, ~6.0 pM). Inhibitor titrations were performed in triplicate as follows: 2.5 µL of the inhibitor stock was added to a 5 µL solution of (unactivated/active) kinase(s) and FRET substrate, followed by 2.5 µL of ATP to start the assay.

FRET-based kinase data was processed in two steps. First, the ratio of the coumarin:fluorescein emission intensities for each well were calculated (445 nm / 520 nm) which was then used to calculate the % phosphorylation using Equation 1. In order to apply equation 1, the 0% and 100% phosphorylation controls were used to establish the minimum and maximum emission ratio values on each assay plate. The 0% phosphorylation control was obtained from a mock kinase reaction run in the absence of ATP. The 100% phosphorylation control was obtained from a mock kinase reaction containing fully phosphorphorylated control peptide. The percent phosphorylation was calculated using the following equation:

(1) $$\%\mathrm{Phosphorylation}=\left(1-\frac{\left(\mathrm{Em}.\mathrm{Ratio}\times F_{100\%}\right)-C_{100\%}}{\left(C_{0\%}-C_{100\%}\right)+\left[\mathrm{Em}.\mathrm{Ratio}\times \left(F_{100\%}-F_{0\%}\right)\right]}\right)\times 100$$

in which Em. ratio is the ratio of coumarin/fluorescein emission signal intensities from sample wells; C_100%_ is the average coumarin emission signal intensity of the 100% phosphorylation control; C_0%_ is the average coumarin emission signal intensity of the 0% phosphorylation control; F_100%_ is the average fluorescein emission signal intensity of the 100% phosphorylation control; and F_0%_ is the average fluorescein emission signal intensity of the 0% phosphorylation control. For the RAF, MEK, and ERK FRET-based assays, the average emission ratio ( ± standard deviation) obtained for the 0% and 100% phosphorylation control reactions from >10 experiments was 5.89 ± 0.15 and 0.34 ± 0.03, respectively. The resulting maximum signal to basal ratio determined from >10 experiments was >17.

### Direct TR-FRET Assay

Development of the TR-FRET assay for RAF using a fluorescein-MEK1 substrate and terbium-labeled phospho-[Ser 217/221] MEK1 antibody has been described [22]. Briefly, 200 nM of fluorescein-MEK1 was used as a substrate in a 10 µL assay. Reactions were conducted in the same 384-well plate type and kinase buffer as described for the FRET assays, initiated by the addition of ATP, and allowed to proceed for 1 hour at RT. After one hour, a 10 µL solution of EDTA and terbium-labeled phospho-[Ser 217/221] MEK1 antibody in 20 mM Tris, pH 7.5 and 0.01% NP-40 was added for a final well volume of 20 µL with final concentrations of antibody and EDTA at 2 nM and 10 mM, respectively. Following a 1 hour incubation at RT, plates were read as described previously [22]. For TR-FRET-based assays, the average maximum signal to basal ratio ( ± standard deviation) obtained using eight different kinases was 8.9 ± 1.0. Inhibition assays were performed by adding 2.5 µL of inhibitor to a 5 µL solution of kinase and substrate, followed by 2.5 µL of ATP. For inhibitor experiments, the final ATP concentration was 100 µM, while the final RAF concentrations were equal to the ~ EC_50_ in the absence of inhibitor (B-RAF, 730 pM; B-RAF V599E, 49 pM; C-RAF, 220 pM). The inhibitor studies were conducted in quadruplicate.

## RESULTS 

**Fig. (1):**
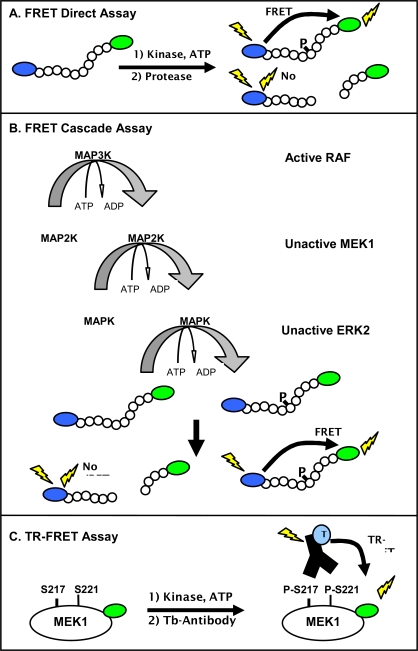
Schematic of fluorescent assay formats used to characterize kinase inhibitors within the RAF-MEK-ERK pathway. (**A**) The FRET direct kinase assay uses a peptide substrate terminally labeled with a coumarin-fluorescein FRET pair and measures the amount of phosphorylated product due to a decrease in sensitivity of the phosphorylated peptide to proteolysis. Proteolysis of the non-phosphorylated product decreases the FRET, while FRET is maintained in the phosphorylated peptide. (**B**) The FRET triple cascade kinase assay detects phosphorylation of a labeled peptide substrate, as described for a direct assay. However, the kinase reaction components vary. In the case of the triple cascade assay shown, the kinase reaction components are active MAP3K kinase (RAF), unactivated MAP2K (MEK1), unactivated MAPK (ERK2), and the FRET peptide substrate. (**C**) The TR-FRET format detects association between a fluorescein-labeled, phosphorylated MEK1 protein and a terbium-labeled phospho- [Ser 217/221] MEK1 antibody. RAF activity is detected by an increase in the fluorescence intensity of the fluorescein acceptor MEK1 relative to the intensity of the terbium phospho-antibody donor.

Two homogeneous fluorescence-based assay formats were employed in our studies. The FRET-based assay uses a peptide substrate that is labeled at opposite ends with donor (coumarin) and acceptor (fluorescein) fluorophores (Fig. **1A**). The substrate is engineered to contain a proteolytic cleavage site proximal to the phosphorylation site, which results in protection of the phosphorylated peptide relative to the non-phosphorylated peptide from proteolysis [21]. In this assay, the primary kinase reaction is followed by a secondary development reaction, in which the kinase reaction is quenched and the protease reaction initiated. Non-phosphorylated substrate is cleaved, resulting in physical separation of the fluorophors and a corresponding decrease in FRET.

Using this FRET-based method, cascade and direct assays were configured in order to recapitulate the RAF-MEK-ERK signaling pathway *in vitro* and facilitate the identification of compounds that interact at allosteric and orthosteric (ATP competitive) sites. The cascade assays utilize an active upstream kinase in combination with unactivated down-stream kinase(s) and an appropriate FRET peptide substrate, which is specifically phosphorylated by the terminal down-stream kinase ERK (Fig. **1B**). In the case of the RAF pathways, active B-RAF, B-RAF V599E, or C-RAF was used with unactivated MEK1, unactivated ERK2, and an ERK-specific peptide substrate; these assays are referred to as triple cascades due to the presence of three kinases. To allow further interrogation of the RAF-MEK-ERK pathway, a double cascade assay was developed using active MEK1 and unactivated ERK2. These cascade assays in combination with a direct ERK2 assay forms the foundation for the RAF pathway assays.

For efficient catalysis, many serine/threonine kinases require kinase/substrate interactions that can not be effectively mimicked by peptide substrates, and we were unsuccessful in developing a FRET peptide-based substrate that would be directly phosphorylated by the RAF family of kinases. In order to fully interrogate the RAF pathway, a direct TR-FRET assay was used that employs MEK1, a physiologic substrate for the RAF family. This assay depends on the binding of a terbium-labeled phospho-[Ser 217/221] specific antibody to a fluorescein labeled MEK1 (Fig. **1C**). Proximity-dependent FRET between the terbium-labeled antibody and the fluorescein labeled phosphorylated-MEK1 can be measured in a time-gated (or “time resolved”) manner.

### Development of Direct and Cascade FRET-Based Assays

**Fig. (2):**
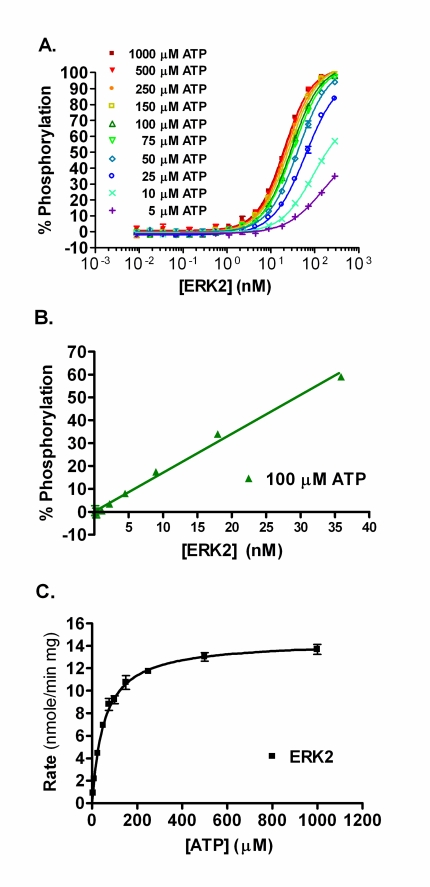
ERK2 Direct FRET Assay. (**A**) The % phosphorylation achieved with increasing active ERK2 in a direct assay using various ATP concentrations. (**B**) Linear plot of the percent phosphorylation achieved for the ERK2 direct assay with 100 µM ATP, with R^2^ ≥ 0.99. (**C**) The % phosphorylation was converted to rate (nmole/min/mg) and plotted versus ATP concentration in order to determine the V_max_ and ATP K_m_^app^ (14.5 nmole/min/mg and 54 µM, respectively). All data points are the average of duplicate determinations.

Using active ERK2 in a direct FRET-based assay and increasing the concentration of either ERK2 or ATP, results in increased phosphorylation of the peptide substrate (Fig. **2A**). The percent phosphorylation for each data point was calculated and the concentration of ERK2 that resulted in ~50% phosphorylation of the substrate at 100 µM ATP was ~29 nM. Increasing the concentration of ERK2 in the assay results in a linear increase in the percent phosphorylation of the substrate achieved up to ~50% (Fig. **2B**). From this data, an ATP K_m_ apparent value of 54 µM was determined for ERK2 (Fig. **2C**).

**Fig. (3):**
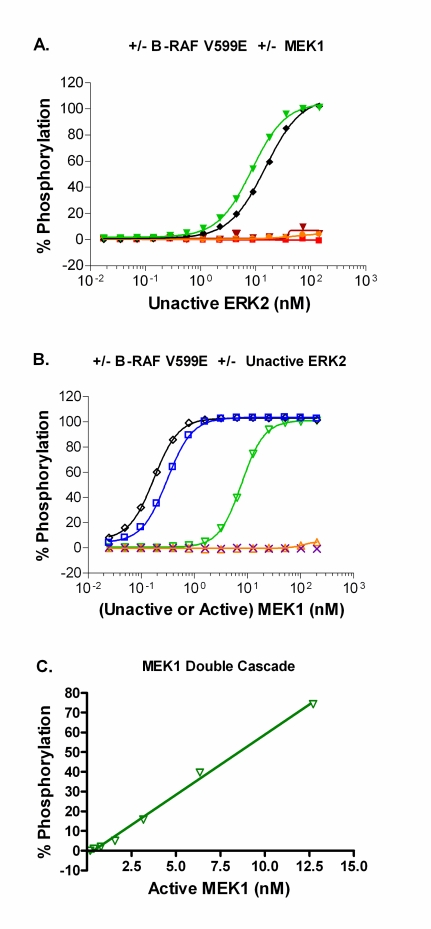
(**A**) Determination of the amount of the unactivated downstream kinase (ERK2) that leads to complete phosphorylation of the peptide substrate in the presence of: No B-RAF V599E and 200 nM active MEK1 (▼, double cascade); 150 nM (10 µg/ml) active B-RAF V599E and 200 nM unactivated MEK1 (♦, triple cascade); No B-RAF V599E and No MEK1 (■); No B-RAF V599E and 200 nM unactivated MEK1 (•); or 150 nM (10 µg/ml) active B-RAF V599E and No MEK1 (▼). (**B**) Determination of the amount of activated MEK1 that leads to complete phosphorylation of the peptide substrate in the presence of: No B-RAF V599E and 140 nM unactivated ERK2 (▽, Double cascade); ~150 nM active B-RAF V599E and 140 nM unactivated ERK2 (□); ~150 nM active B-RAF V599E and No ERK2 (×). Determination of the amount of unactivated MEK1 that leads to complete phosphorylation of the peptide substrate in the presence of: ~150 nM active B-RAF V599E and 140 nM unactivated ERK2 (◇, triple cascade); No B-RAF V599E and 140 nM unactivated ERK2 (Δ). (**C**) Linear plot of the percent phosphorylation achieved for the double cascade with increasing concentration of active MEK1, using the optimized concentration for unactive ERK2 (140 nM), with R^2^ ≥ 0.99. All data points represent the mean ± standard deviation from triplicate samples.

In order to develop the cascade assays, we took a multistep approach that involved the sequential optimization of unactivated and active kinase concentrations in the reactions. First, the amount of the unactivated ERK2 required for complete phosphorylation of the peptide substrate in the presence of excess upstream kinase(s) was determined. For the double cascade the upstream kinase is active MEK1, while for the triple cascades active RAF and unactivated MEK1 are the upstream kinases used. In the double cascade, complete phosphorylation of the substrate is achieved when 200 nM (~10 µg/mL) active MEK1 was used with 140 nM (~10 µg/mL) of unactivated ERK2 (Fig. **3A**). For the B-RAF V599E triple cascade, complete phosphorylation of the substrate is achieved when 150 nM (~10 µg/mL) active B-RAF V599E and 200 nM (~ 10 µg/mL) unactivated MEK1 were used with 140 nM of unactivated ERK2 (Fig. **3A**). As expected, no significant phosphorylation of substrate is achieved under conditions lacking the full activation sequence for the RAF-MEK-ERK pathway: unactivated ERK2 alone; unactivated MEK1 plus unactivated ERK2; or active RAF plus unactivated ERK2 (Fig. **3A**). Similar results were obtained when B-RAF and C-RAF were used instead of B-RAF V599E (data not shown). Therefore, a concentration of 140 nM unactivated ERK2 was selected for subsequent experiments.

After defining the optimal concentration of unactivated ERK2 to use in the assay, we then optimized the concentration of MEK1. In the case of the double cascade, active MEK1 was titrated with 140 nM unactivated ERK2 and ~50% phosphorylation of the substrate was seen with ~8 nM active MEK1 (Fig. **3B**). For the triple cascades, unactivated MEK1 was titrated in the presence of 140 nM unactivated ERK2 plus 10 µg/mL RAF. For the B-RAF V599E triple cascade, complete phosphorylation of the substrate was achieved at a concentration of unactivated MEK1 greater than ~6 nM (Fig. **3B**). These results are similar to that observed when active MEK1 is included in the assay rather than unactivated MEK1 (Fig. **3B**). This observation suggests that B-RAF V599E is able to fully activate the unactivated MEK1 preparation, and that there is a small portion of the active MEK1 preparation that is unactivated but that can be activated by B-RAF V599E during the one hour kinase reaction. As expected, no significant phosphorylation of substrate is achieved under conditions lacking the full activation sequence for the RAF-MEK-ERK pathway (Fig. **3B**). Similar results were obtained with B-RAF or C-RAF (data not shown). The concentration of unactivated MEK1 selected for subsequent use in the three triple cascade assays was 20 nM.

**Fig. (4):**
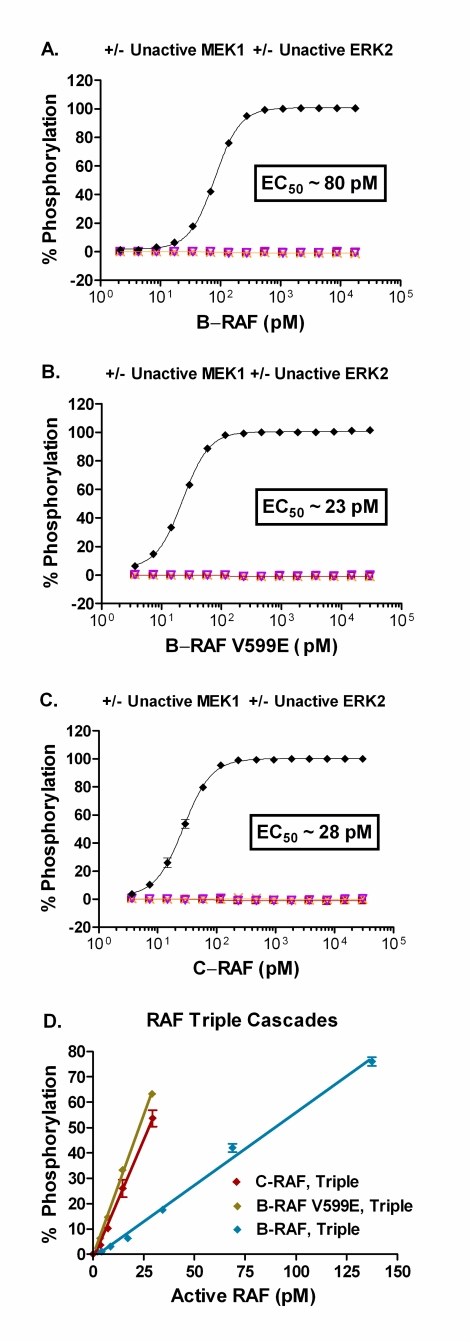
Using the optimal concentration of unactivated ERK2 of 140 nM (~10 µg/ml) and unactivated MEK1 of 20 nM (~1 µg/ml), determination of amount of active B-RAF V599E to use for subsequent kinase inhibitor studies (♦, triple cascade). Control reactions lacking a portion of the RAF-MEK-ERK pathway were: titration of B-RAF V599E without MEK1 and without ERK2 (□); titration of B-RAF V599E with 140 nM unactivated ERK2 without MEK1 (▽); titration of B-RAF V599E with 20 nM unactivated MEK1 without ERK2 (×). (D) Linear plots of the percent phosphorylation achieved for each of the triple cascades with increasing concentrations of active RAF isoform, using the optimized concentrations for unactive MEK1 (20 nM) and unactive ERK2 (140 nM), with R^2^ ≥ 0.99. All data points represent the mean ± standard deviation from triplicate samples.

The third step in the assay optimization process was to titrate each RAF isoform against unactivated MEK1 and ERK2 at the optimal concentration of each, as determined above (Fig. **4**). For the triple cascade assays EC_50 _values of 80 pM, 23 pM, and 28 pM were obtained for B-RAF, B-RAF V599E, and C-RAF, respectively (Fig. **4A-C**). As expected, no significant phosphorylation of substrate was observed under conditions lacking the full activation sequence for the RAF-MEK-ERK pathway (Fig. **4A-C**). Increasing the concentration of an active RAF in the triple cascade assays (Fig. **4D**) or active MEK1 in the double cascade assay (Fig. **3C**) resulted in a linear increase in the percent phosphorylation of the substrate achieved. The Z’ value was ≥ 0.5 at substrate phosphorylation levels greater than ~5 – 10% (data not shown). Therefore, subsequent experiments utilized concentrations of active RAFs or active MEK1 that resulted in 10 – 50% phosphorylation of the peptide substrate.

### TR-FRET Direct Assay 

**Fig. (5):**
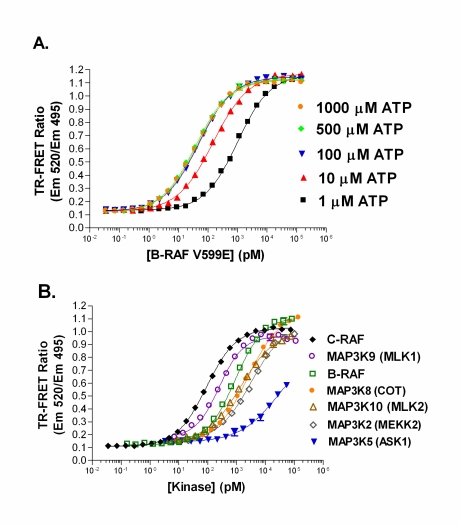
Direct TR-FRET MAP3K assays. (**A**) The TR-FRET ratios (Em 520 nm/Em 495 nm) resulting from titrations B-RAF V599E with one of five different ATP concentrations (1 – 1000 µM). (**B**) The TR-FRET direct assay was utilized with seven different MAP3Ks using a final ATP concentration of 500 µM. All data points represent the mean ± standard deviation from triplicate samples.

Using a native substrate for RAF (*i.e.* MEK1), a direct TR-FRET assay for C-RAF has been recently reported [22]. In this study, RAF titrations revealed that at ATP concentrations between 1 – 1000 µM, B-RAF V599E was the most active, followed by C-RAF, and then B-RAF; EC_50_ values at 100 µM ATP were 49 pM for B-RAF V599E, 220 pM for C-RAF, and 730 pM for B-RAF (Fig. **5A** for B-RAF V599E). This is the same order of activity observed for the RAF isoforms in the FRET-based triple cascade assays. The Z’ value was ≥ 0.7 when the EC_30_ or higher concentrations of C-RAF were used (data not shown). The fluorescein-MEK1 and terbium-labeled phospho-[Ser217/221] MEK1 antibody assay also serves as a good method to quantify activity of other MAP3K’s beyond the RAFs (Fig. **5B**).

### Inhibition of RAF-MEK-ERK Cascade and Direct Inhibition of MEK

**Fig. (6):**
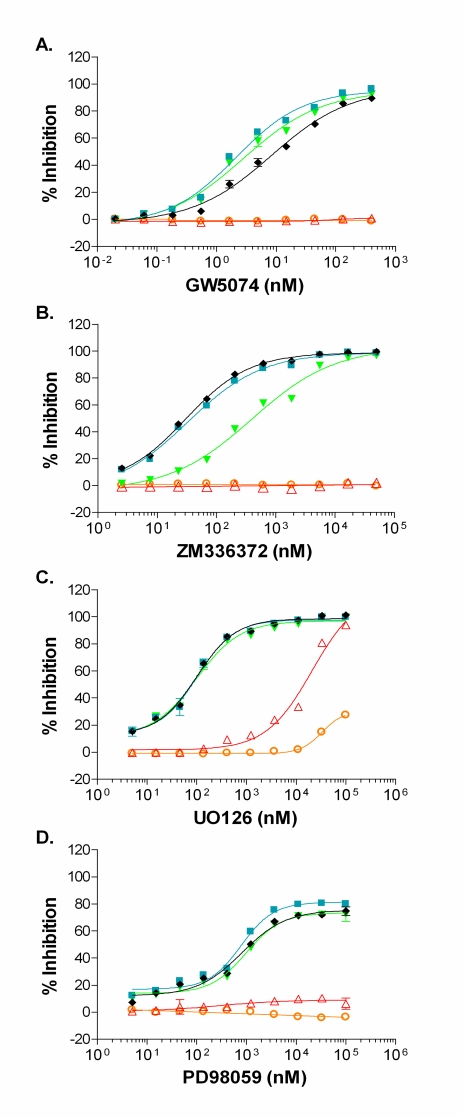
Inhibitor titrations are shown for the FRET assays: ERK2 direct (O); MEK1-ERK2 double cascade (Δ); B-RAF-MEK1-ERK2 triple cascade (▼); B-RAF V599E-MEK1-ERK2 triple cascade (■); and C-RAF-MEK1-ERK2 triple cascade (♦). For all assay formats, concentrations of active and unactivated enzymes were optimized in order to achieve 10 - 50% phosphorylation of the FRET substrate in the presence of 100 µM ATP and the absence of inhibitor. Two of the compounds tested are known to inhibit RAF activity, GW5074 (**A**) and ZM336372 (**B**). The other two compounds are allosteric MEK inhibitors: U0126 (**C**) can differentially inhibit MEK activity as well as MEK activation, while PD98059 (**D**) inhibits RAF phosphorylation of MEK but not MEK activity. All data points represent the mean ± standard deviation from quadruplicate samples.

Cascade assays offer the potential to identify either allosteric or ATP competitive compounds that target unactivated or active kinases within the recapitulated kinase signaling pathway, but with this advantage comes the necessity for a means to identify (deconvolute) the site of action for the active compound. Using a small set of reference inhibitory compounds, we used our abbreviated cascade and direct assays as a means to deconvolute inhibition within the RAF pathway. Staurosporine, a broad based ATP-competitive kinase inhibitor, had an IC_50_ of ~4 nM in the double cascade assay and an IC_50_ that is ~10-fold larger in the triple cascades assaying B-RAF, B-RAF V599E, or C-RAF (Table **1**). A portion of this difference may be accounted for by the molar difference in MEK1 content, which is ~ 4 nM in the double cascade assay and ~20 nM in the triple cascade assay. Staurosporine was less effective at inhibiting the ERK2 direct assay, with an ~70-fold larger IC_50_ than that observed for the triple cascades (Table **1**). These results for staurosporine indicate that differential inhibition of kinases within the pathway can be detected and that IC_50_ values for potent inhibitors may be limited by the concentration of kinase present in the reaction. GW5074, a known RAF1 inhibitor, displayed complete inhibition of the three RAF triple cascade assays, but displayed no inhibition of either the ERK2 direct or MEK1-ERK2 double cascade assays (Fig. **6A**). The 9 nM IC_50_ determined in the triple C-RAF cascade (Table **1**) is in close agreement with the published IC_50_ of 9 nM for GW5074 of C-RAF [23]. As expected, GW5074 inhibited B-RAF, B-RAF V599E, and C-RAF in the direct TR-FRET assay with rank order IC_50_ values in agreement with the triple cascade assays (Table **1**), but with absolute IC_50_ values 2.5 to 5- fold higher than seen in the cascade assays. ZM336372 has been reported to inhibit C-RAF at 10-fold lower IC_50_ relative to B-RAF [24] and the FRET-based assay results were consistent with this finding: ~13-fold lower IC_50_ observed for the C-RAF triple cascade relative to the B-RAF triple cascade and no inhibition of either the ERK2 direct or MEK1 double cascade (Fig. **6B**, Table **1**). The RAF direct TR-FRET assays confirmed that ZM336372 acts by inhibiting RAF activity and also confirmed a preference for C-RAF inhibition relative to B-RAF, as a ~6-fold decrease in the IC_50_ for C-RAF was found relative to B-RAF. ZM336372 was equally potent for C-RAF and B-RAF V599E in the triple cascades (Fig. **6B**), while ZM336372 had a ~3-fold lower IC_50_ for C-RAF relative to B-RAF V599E in the RAF TR-FRET direct assay.

U0126 is an inhibitor that has a unique mode of action in that it binds directly to a site that is non-competitive with ATP (an allosteric site) on MEK1 that prevents phosphorylation by RAF; it can bind both unactivated and active MEK1/2, but it is a more potent inhibitor of RAF phophorylation of MEK1/2 [25-27]. As expected for an inhibitor of RAF phophorylation of MEK1, U0126 was able to inhibit the three RAF triple cascades with near equal potency, ~65 nM (Fig. **6C**, Table **1**). A >100-fold decrease in potency was observed for the MEK1 double cascade and ERK2 direct assays relative to the three triple cascade assays (Table **1**). The IC_50_ for the triple cascade assays is comparable to the ~70 nM IC_50_ observed for U0126 inhibition of MEK1 activation observed in Swiss 3T3 cells, while the >10 µM potency observed for the MEK1 double cascade is consistent with the 13 µM IC_50_ reported for U0126 inhibition of MEK1 [27]. The suggestion from our data for a compound with unknown mode of action is that the potent level of inhibition (~65 nM) likely is for RAF activity and/or MEK1 activation, while the low inhibitory activity (µM potency) is likely for active MEK1. U0126 had very weak inhibitory activity in the B-RAF, B-RAF V599E, and C-RAF TR-FRET direct assays (Table **1**). Therefore, the TR-FRET data suggests that the potent U0126 inhibitory activity observed in the triple cascade is specific for unactivated MEK1, not for active RAF. Furthermore, the fact that U0126 does not strongly inhibit the C-RAF TR-FRET direct assay suggests that the fluorescein labeling of MEK1 sterically blocks the MEK1 allosteric site that binds U0126.

PD98059 is an allosteric MEK inhibitor that has been reported to inhibit MEK1 activation, but not MEK1 activity [25, 27, 28]. Our results showing that the three RAF triple cascades have similar IC_50_ values and that no inhibition was observed for the MEK1 double cascade or ERK2 direct assays were consistent with this mechanism of action (Fig. **6D**, Table **1**). PD98059 did not inhibit the B-RAF, B-RAF V599E, or C-RAF direct assays (Table **1**). Again, the TR-FRET data would confirm that the inhibitory activity observed in the triple cascade was specific for unactivated MEK1, not for active RAF.

**Fig. (7):**
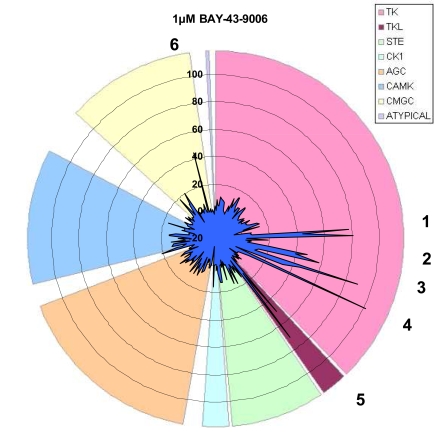
Radial plot of the inhibition of 1 µM BAY-43-9006 against 231 kinases using the FRET-based assays. For each of the six areas numbered, kinases represented within each area and the percent inhibition observed are listed. Area 1: RET (77%), RET V804L (44%), and RET Y791F (80%). Area 2: VEGFR2 (48%), VEGFR3 (40%), and FMS (40%). Area 3: FLT3 (89%), FLT3 D835Y (45%), and PDGFR α (33%). Area 4: PDGFR α V561D (101%). Area 5: C-RAF (50%), B-RAF (13%), and B-RAF V599E (71%). Area 6: HIPK4 (45%).

By determining a compound’s inhibitory profile against a series of kinases, alternate targets that may be valuable for therapeutic intervention or deleterious due to off-target interactions can be revealed. Using the FRET-based assay technology described, we have developed assays for over 230 kinases. We used these assays to generate an inhibition profile for the RAF inhibitor BAY-43-9006 (Fig. 7). As expected, B-RAF, B-RAF V599E, and C-RAF were inhibited and the inhibition was more pronounced using 10 µM vs. 1 µM BAY-43-9006 (91% vs. 50%, 44% vs. 13%, and 93% vs. 71% inhibition, respectively). Other kinases reported to be inhibited by BAY-43-9006, were also detected using 1µM BAY-43-9006: VEGFR2 (48%), VEGFR3 (40%), FLT3 (89%). Additional kinases on the 231 panel that displayed ≥ 40% inhibition by 1 µM BAY-43-9006 were: RET (77%), RET Y791F (80%), RET V804L (44%), FMS (58%), PDGFR α V561D (101%), HIPK1 (45%), and FLT3 D835Y (45%). Interestingly, despite complete inhibition of PDGFR α V561D using 1 µM BAY-43-9006, increasing the concentration to 10 µM BAY-43-9006 resulted in no inhibition of PDGFR α D842V mutant (3%), weak inhibition of PDGFR α T674I mutant (37%), and moderate inhibition of PDGFR α (75%).

## DISCUSSION

The RAF/MEK/ERK signaling pathway is ubiquitious in eukaryotic cells and regulates numerous physiologic processes such as cellular proliferation, differentiation, and survival [29]. The over-activation of RAF due to dysregulated signaling upstream of RAF or due to mutations within RAF are involved in numerous stages of cancer, including tumor formation, angiogenesis, metastasis, and resistance to therapeutic regiments [14]. As a result, the RAF pathway has been targeted in drug discovery. We developed two assay formats (FRET-based and TR-FRET-based) that allow interrogation of steps within the RAF-MEK-ERK signaling pathway using a combination of cascade and direct assay formats. We have found that the ratiometric method for FRET data analysis dramatically reduces well-to-well variability, which contributes to the excellent Z’ values (≥ 0.5) we observed, even under reaction conditions that produced low percent phosphorylation. We show here that a traditional plot of the ATP concentration vs. the rate of product formation can be employed to determine the ATP K_m_ for a kinase in the FRET direct format. In addition, we have now successfully recapitulated the RAF-MEK-ERK pathway using a triple cascade FRET approach.

For the FRET and TR-FRET assays, we were able to determine IC_50_ values for several reference compounds. The known RAF1 inhibitor, GW5074, had IC_50_ values for the RAF triple cascade assays and RAF direct TR-FRET assays of 2 - 9 nM and 5 - 24 nM, respectively (Table **1**). These values are in agreement with the published GW5074 IC_50_ value of 9 nM for C-RAF [23]. A second known RAF1 inhibitor, ZM336372, has been shown to have an IC_50_ of 70 nM for C-RAF [24]; we obtained similar IC_50_ values for C-RAF using the FRET and TR-FRET assays (30 nM and 90 nM, respectively). ZM336372 is reported to have 10-fold selectivity for C-RAF over B-RAF and we observed ~6-13-fold selectivity using the FRET and TR-FRET assays. U0126 has a weak ability to inhibit MEK1 with an IC_50_ of 13 µM, but an ~100-fold preferential inhibition of MEK1 activation determined *via *radiometric kinase assays [27]. This is in agreement with the >10 µM IC_50_ we observed for the double cascade MEK1 FRET-based assay, and the ~65 nM IC_50_ observed for the FRET-based RAF triple cascades. PD98059 inhibits MEK1 activation by RAF, but not MEK1 activity [25, 27, 28]. Using ~300 nM MEK1 as a substrate, PD98059 inhibited the activation of MEK1 by C-RAF with an IC_50_ of 2-7 µM [28]. Our FRET-based assay results are consistent with the reported mechanism of action for PD98059, but the IC_50_ observed in the FRET triple cascade assays were substantially lower, ~300-420 nM. The difference may be partially due to the ~15-fold lower concentration of MEK1 (20 nM) used in the FRET-based triple cascade assays compared to the previous report (300 nM MEK1).

The successful development of FRET-based assays was dependent on the ability to design a peptide substrate for a given kinase that also contains a protease cleavage site proximal to the phosphorylation site. Since the original report documenting this approach to kinase assays [21], we have developed assays for over 230 kinases utilizing a set of 31 peptide substrates. This set of assays is useful for compound profiling, as was demonstrated using the multi-kinase inhibitor BAY-43-9006. Using this approach, we were able to identify kinases that are known to be inhibited by BAY-43-9006, including C-RAF, B-RAF V599E, VEGFR2, VEGFR3, and FLT3. Recently, BAY-43-9006 has shown potent inhibition of RET, RET V804L, and RET V804M, which are associated with medullary and papillary thyroid carcinomas [30]. Our results extend this work, and demonstrate more potent inhibition of the RET mutant Y791F relative to RET V804L mutant. BAY-43-9006 has also been reported to inhibit the oncogenic fusion protein of FIP1-like protein and PDGFRα that causes eosinophilic leukemia; the T674I mutant of this fusion protein is imatinib-resistant [31]. We demonstrate inhibition of the isolated PDGFRα T674I mutant kinase and show that the PDGFRα V561D mutant is more sensitive to BAY-43-9006 inhibition, while the PDGFRα D842V mutant is insensitive to BAY-43-9006. Both the PDGFRα V561D and PDGFRα D842V mutants are activating mutations found in gastrointestinal tumors [32]. Our profiling results may indicate a use for BAY-43-9006 in patients bearing PDGFRα V561D mutants, but not in patients bearing PDGFRα D842V mutants.

Traditional radiometric RAF/MEK/ERK cascade assays have been reported [33], and several reports have been recently published describing fluorescent methods to target the RAF signaling pathway [34, 35] or the cancer Osaka thyroid (COT) signaling pathway [36, 37]. Mallon and colleagues described a cascade utilizing active C-RAF, unactivated MEK1, and unactivated ERK2 in which phosphorylated ERK2 was detected using a europium labeled antibody in a non-homogenous DELFIA format [34]. In this assay, the RAF kinase cascade assay was conducted in 96-well plates; the stopped reactions were transferred to assay plates to allow capture of the GST-ERK2 substrate, and washing/incubation steps occurred with the phospho-ERK2 antibody, and with the europium-labeled secondary antibody. These authors employed secondary assays for measuring RAF phosphorylation of MEK1 *via *western blot, and for measuring MEK1 activity *via *an assay similar to their RAF cascade, except RAF was eliminated and active MEK1 was used. Although successful for the compound characterization, the non-homogenous nature of this assay as well as the secondary assays (involving plate transfer and washing) is cumbersome and increases potential for error in the drug discovery process. Newbatt and colleagues described a non-homogeneous europium-based DELFIA cascade assay utilizing active B-RAF V599E, unactivated MEK1, unactivated ERK2, and ELK1 as the substrate for ERK2 [35]. The B-RAF V599E kinase cascade assay was conducted in 384-well plates in which the ELK1 substrate was coated, washed and blocked prior to conducting the kinase reaction. Following the kinase reaction, the plates were washed, incubated with a combination of primary phospho-ELK1 and europium-labeled secondary antibodies, and finally washed again prior to addition of the enhancement solution. To understand the point of action for inhibitors within the cascade assay, Newbatt and colleagues employed two non-homogeneous secondary assays: (1) an activated MEK1 assay using unactivated ERK2 and ELK1 as a substrate, analogous to the RAF cascade; and (2) an activated B-RAF V599E direct assay using GST-MEK1, glutathione coated plates, anti-phosho-MEK antibody, and europium-labeled secondary antibody [35]. The work of Newbatt *et al. *[35] produces some improvements over the assays used by Mallon *et al. *[34]; these include miniaturization to 384-well plates and elimination of the transfer step. However, the necessity to coat the substrate onto the assay plate, wash & block the plates prior to the kinase assay, and wash the assay plate after addition of antibodies adds significant amounts of labor and increased opportunity for error. Jia and colleagues described the use of a homogeneous time-resolved fluorescence (HTRF) cascade format for COT, C-RAF, and MEK1 utilizing biotinylated-myelin basic protein (MBP) as the terminal substrate [36]. The assay sequence involved the following: (1) 40 µL kinase reactions in 96-well half-volume plates; (2) addition of stop reagent; (3) addition of europium labeled anti-phopho-MBP antibody and allophycocyanin-labeled streptavidin; (4) overnight incubation at 4°C. The homogenous time-resolved nature of this assay is an improvement over previous work, but struggles from the use of an overnight incubation at 4°C and a 96-well plate format.

The RAF and MEK1 cascade format FRET-based assays that we describe offer significant improvements for reagent use, time, and labor. The assays we have developed are conducted as 10 µL kinase reactions in 384-well plates, and require only two sequential one hour incubations at room temperature. The FRET cascade, as well as the direct, assays offer the ability to quantify the level of phosphorylation achieved on each assay plate by simple inclusion of three controls (a no inhibitor control, a 0% phosphorylation control, and a synthetic 100% phosphorylation control), thereby ensuring that the assay was conducted within the linear range of the kinase reaction. The Z’ values obtained for the FRET cascade assays were ≥0.5 at <10% phosphorylation, indicating that a robust inhibitor screen could be run at low levels of phosphorylation. The FRET-based and TR-FRET-based secondary assays we employed to interrogate the location of inhibitor action were also homogeneous, required two sequential one hour room temperature incubations, and resulted in assays with Z’ ≥ 0.5. Both the FRET-based and TR-FRET-based kinase assay formats utilized a ratiometric method for data analysis that reduced well-to-well variability. In addition to these common advantages for the FRET-based and TR-FRET based assays, there are several advantages that are unique to either format. A specific benefit of the TR-FRET assay we employed is the inherent resistance to common forms of assay interference such as colored, fluorescent, or precipated compounds [22]. Although these forms of interference can be a problem for non-time-resolved fluorescent assays, they can be mitigated for in the FRET-based assay by running control reactions with compound and assessing the effect on the fluorescent emissions. A specific benefit of the FRET-based approach is the ability to develop kinase assays without an antibody. The FRET-based cascade assays also offer the potential to develop assays for kinases that require binding to the native substrate’s tertiary structure and thereby allow the identification of allosteric inhibitors for kinases within the cascade.

Recently, various COT kinase assay formats were compared for the purpose of identifying an assay suitable for finding inhibitors of COT activity [36]. Using three direct COT assays that each had a different substrate, the authors concluded that use of the unactivated MEK1 protein as a substrate can result in the identification of compounds that inhibit COT by binding to MEK1 rather than by specifically inhibiting COT. These compounds (similar to PD98059 or U0126) were not valuable to these authors since they were seeking compounds that specifically interacted with COT. In our direct C-RAF TR-FRET assay, we employed a fluorescein-labeled-unactivated-MEK1 to assess inhibitors of C-RAF activity. Using this assay, neither U0126 nor PD98059 significantly inhibited C-RAF, while the known RAF inhibitors GW5076 and ZM336372 produced IC_50_ values consistent with literature values. An explanation for the lack of inhibition by U0126 & PD98059 is that one of the fluoresceins that label MEK1 sterically blocks the MEK1 allosteric site that binds U0126 and PD98059. This is an advantage for researchers interested in identifying compounds that specifically inhibit MAP3K by binding to MAP3K rather than by binding to MEK. Kinase titrations for other MAP3K’s using fluorescein-unactivated-MEK1 as a substrate in the direct TR-FRET assay are shown in Fig. (**5B**). For researchers interested in compounds that act within the RAF-MEK-ERK pathway and possibly through allosteric binding sites such as those of MEK1 that are bound by PD-0325901 or ARRY-142886, the cascade approach is desirable. Deconvolution of the triple cascade can readily be obtained using high-throughput compatible double cascade and direct assays, as demonstrated here.

## CONCLUSION

The RAF/MEK/ERK signaling pathway forms a critical link between extra-cellular stimuli and intra-cellular responses that ultimately regulate cellular proliferation, differentiation, mobility, and survival. Mutation of RAF or over-activation of RAF *via *aberrant upstream signaling impacts numerous stages of tumorigenesis. The series of assays we have developed target the RAF/MEK/ERK pathway and allow the identification of compounds that act in an orthosteric or allosteric manner as well as the deconvolution of the inhibitory activity. These fluorescent assays utilize a homogenous low volume high-throughput format, employ ratiometric data analysis leading to decreased well-to-well variability, can be conducted at low substrate conversion with Z’ ≥ 0.5, and require only two sequential one hour room temperature incubations. As the benefit to knowing a compound’s inhibitory potential against multiple kinases grows, the need to determine the inhibition profile expands. Since the FRET-based assay format has been successfully applied to over 230 kinases, it is well suited for compound profiling. In summary, the identification of RAF/MEK/ERK pathway inhibitors hold the potential to have clinical impact and facile methods should enable further drug discovery in this pathway, and the assays that we have described should aid in those efforts.

## Figures and Tables

**Table 1.** Inhibitor IC_50_ Values for Direct and Cascade Kinase Assays. Kinase Reactions were Conducted in the Presence of Increasing Inhibitor Concentrations for the FRET Direct, FRET Cascades, and TR-FRET Direct Assays Using 100 μM ATP

		Inhibitor IC_50_ (nM)				
Format	Assay	Staur.	GW5074	ZM336372	U0126	PD98059
FRET Direct	ERK2	4,400	>400	>50,000	>50,000	>50,000
FRET Double	MEK1-ERK2	4	>400	>50,000	>10,000	>50,000
FRET Triple	BRAF-MEK1-ERK2	56	3	400	65	420
FRET Triple	BRAF V599E-MEK1-ERK2	69	2	32	65	300
FRET Triple	CRAF-MEK1-ERK2	63	9	30	66	380
TR-FRET Direct	BRAF	>50,000	15	580	>40,000	>50,000
TR-FRET Direct	BRAF V599E	>50,000	4.9	270	≥5,000	>50,000
TR-FRET Direct	CRAF	>50,000	24	90	>50,000	>50,000

## ABBREVIATIONS

FRET: = Förster-resonance energy transfer
MAPK: = Mitogen-activated protein kinase
MAP2K: = Mitogen-activated protein kinase kinase
MAP3K: = Mitogen-activated protein kinase kinase kinase
RTK: = Receptor tyrosine kinase
TR-FRET: = Time-resolved Förster-resonance energy transfer

## References

[1] Widmann C, Gibson S, Jarpe MB, Johnson GL (1999). Mitogen-activated protein kinase: conservation of a three-kinase module from yeast to human. Physiol Rev.

[2] Hoshino R, Chatani Y, Yamori T, Tsuruo T, Oka H, Yoshida O (1999). Constitutive activation of the 41-/43-kDa mitogen-activated protein kinase signaling pathway in human tumors. Oncogene.

[3] Gioeli D, Mandell JW, Petroni GR, Frierson HF Jr, Weber MJ (1999). Activation of mitogen-activated protein kinase associated with prostate cancer progression. Cancer Res.

[4] Mandell JW, Hussaini IM, Zecevic M, Weber MJ, VandenBerg SR (1998). In situ visualization of intratumor growth factor signaling: immunohistochemical localization of activated ERK/MAP kinase in glial neoplasms. Am J Pathol.

[5] Marshall CJ (1995). Specificity of receptor tyrosine kinase signaling: transient versus sustained extracellular signal-regulated kinase activation. Cell.

[6] Bos JL (1989). ras oncogenes in human cancer: a review. Cancer Res.

[7] Andreyev HJ, Norman AR, Cunningham D, Oates JR, Clarke PA (1998). Kirsten ras mutations in patients with colorectal cancer: the multicenter "RASCAL" study. J Natl Cancer Inst.

[8] Garnett MJ, Marais R (2004). Guilty as charged: B-RAF is a human oncogene. Cancer Cell.

[9] Wan PT, Garnett MJ, Roe SM (2004). Mechanism of activation of the RAF-ERK signaling pathway by oncogenic mutations of B-RAF. Cell.

[10] Davies H, Bignell GR, Cox C (2002). Mutations of the BRAF gene in human cancer. Nature.

[11] Wellbrock C, Ogilvie L, Hedley D (2004). V599EB-RAF is an oncogene in melanocytes. Cancer Res.

[12] Ikenoue T, Hikiba Y, Kanai F (2004). Different effects of point mutations within the B-Raf glycine-rich loop in colorectal tumors on mitogen-activated protein/extracellular signal-regulated kinase kinase/extracellular signal-regulated kinase and nuclear factor kappaB pathway and cellular transformation. Cancer Res.

[13] Hingorani SR, Jacobetz MA, Robertson GP, Herlyn M, Tuveson DA (2003). Suppression of BRAF(V599E) in human melanoma abrogates transformation. Cancer Res.

[14] Gollob JA, Wilhelm S, Carter C, Kelley SL (2006). Role of Raf kinase in cancer: therapeutic potential of targeting the Raf/MEK/ERK signal transduction pathway. Semin Oncol.

[15] Gimbrone MA Jr, Leapman SB, Cotran RS, Folkman J (1972). Tumor dormancy *in vivo* by prevention of neovascularization. J Exp Med.

[16] Folkman J, Kalluri R (2004). Cancer without disease. Nature.

[17] Wilhelm SM, Carter C, Tang L (2004). BAY 43-9006 exhibits broad spectrum oral antitumor activity and targets the RAF/MEK/ERK pathway and receptor tyrosine kinases involved in tumor progression and angiogenesis. Cancer Res.

[18] Yeh TC, Marsh V, Bernat BA (2007). Biological characterization of ARRY-142886 (AZD6244), a potent, highly selective mitogen-activated protein kinase kinase 1/2 inhibitor. Clin Cancer Res.

[19] Wang JY, Wilcoxen KM, Nomoto K, Wu S (2007). Recent advances of MEK inhibitors and their clinical progress. Curr Top Med Chem.

[20] Zhang JH, Chung TD, Oldenburg KR (1999). A Simple Statistical Parameter for Use in Evaluation and Validation of High Throughput Screening Assays. J Biomol Screen.

[21] Rodems SM, Hamman BD, Lin C (2002). A FRET-based assay platform for ultra-high density drug screening of protein kinases and phosphatases. Assay Drug Dev Technol.

[22] Riddle SM, Vedvik KL, Hanson GT, Vogel KW (2006). Time-resolved fluorescence resonance energy transfer kinase assays using physiological protein substrates: applications of terbium-fluorescein and terbium-green fluorescent protein fluorescence resonance energy transfer pairs. Anal Biochem.

[23] Lackey K, Cory M, Davis R (2000). The discovery of potent cRaf1 kinase inhibitors. Bioorg Med Chem Lett.

[24] Hall-Jackson CA, Eyers PA, Cohen P (1999). Paradoxical activation of Raf by a novel Raf inhibitor. Chem Biol.

[25] Ballif BA, Blenis J (2001). Molecular mechanisms mediating mammalian mitogen-activated protein kinase (MAPK) kinase (MEK)-MAPK cell survival signals. Cell Growth Differ.

[26] Favata MF, Horiuchi KY, Manos EJ (1998). Identification of a novel inhibitor of mitogen-activated protein kinase kinase. J Biol Chem.

[27] Davies SP, Reddy H, Caivano M, Cohen P (2000). Specificity and mechanism of action of some commonly used protein kinase inhibitors. Biochem J.

[28] Alessi DR, Cuenda A, Cohen P, Dudley DT, Saltiel AR (1995). PD 098059 is a specific inhibitor of the activation of mitogen-activated protein kinase kinase *in vitro* and *in vivo*. J Biol Chem.

[29] Kolch W (2000). Meaningful relationships: the regulation of the Ras/Raf/MEK/ERK pathway by protein interactions. Biochem J.

[30] Carlomagno F, Anaganti S, Guida T (2006). BAY 43-9006 inhibition of oncogenic RET mutants. J Natl Cancer Inst.

[31] Lierman E, Folens C, Stover EH (2006). Sorafenib is a potent inhibitor of FIP1L1-PDGFRalpha and the imatinib-resistant FIP1L1-PDGFRalpha T674I mutant. Blood.

[32] Heinrich MC, Corless CL, Duensing A (2003). PDGFRA activating mutations in gastrointestinal stromal tumors. Science.

[33] McDonald OB, Chen WJ, Ellis B (1999). A scintillation proximity assay for the Raf/MEK/ERK kinase cascade: high-throughput screening and identification of selective enzyme inhibitors. Anal Biochem.

[34] Mallon R, Feldberg LR, Kim SC (2001). An enzyme-linked immunosorbent assay for the Raf/MEK1/MAPK signaling cascade. Anal Biochem.

[35] Newbatt Y, Burns S, Hayward R (2006). Identification of inhibitors of the kinase activity of oncogenic V600E BRAF in an enzyme cascade high-throughput screen. J Biomol Screen.

[36] Jia Y, Quinn CM, Clabbers A (2006). Comparative analysis of various *in vitro* COT kinase assay formats and their applications in inhibitor identification and characterization. Anal Biochem.

[37] Han S, Zhou V, Pan S (2005). Identification of coumarin derivatives as a novel class of allosteric MEK1 inhibitors. Bioorg Med Chem Lett.

